# Generating Patient-Specific Anti-Tumor Responses with Non-Genetically Altered ‘Off-the-Shelf’ Allogeneic Cell Therapy: Leveraging Allo-Incompatibility for In Situ Vaccination

**DOI:** 10.3390/vaccines14070619

**Published:** 2026-07-14

**Authors:** Michael Har-Noy

**Affiliations:** Immunovative Therapies, Ltd., Jerusalem 9610203, Israel; harnoy@immunovative.com

**Keywords:** cancer vaccine, immunotherapy, cold-to-hot tumor conversion, immunological cell death (ICD), tumor microenvironment

## Abstract

Background: Generating personalized anti-tumor immune responses remains a primary objective of precision oncology, yet conventional autologous platforms face critical biological and logistical constraints. While current research modifies allogeneic lines to evade host clearance, this perspective outlines a translational framework designed to leverage host-donor incompatibility as an active immunomodulatory asset to remodel the solid tumor microenvironment (TME). The framework proposes expanding a systemic pool of circulating, allo-specific host type 1 helper (Th1) memory cells via iterative intradermal injections of completely mismatched, activated donor Th1 cells, followed by a systemic intravenous rechallenge to provoke a controlled host-versus-graft (HvG) rejection response. Rapid intravascular clearance of donor cells is hypothesized to drive a transient, Type 1 cytokine wave that activates host effector populations via bystander pathways, promoting their extravasation into the tumor stroma to induce immunogenic cell death (ICD). This paradigm is contextualized by Phase 2B data in refractory microsatellite stable (MSS) metastatic colorectal cancer, where a dual-route allogeneic Th1 regimen demonstrated a median overall survival (OS) signal of 16.4 months despite an 89.5% conventional radiological progression rate. Ultimately, this framework provides a predictable, non-engineered conceptual mechanism to elicit a patient-specific adaptive immune response without ex vivo customization.

## 1. Introduction

The primary objective of modern precision oncology remains the generation of a highly personalized, tumor-specific adaptive immune response capable of eradicating established solid malignancies and establishing long-term immunological memory. To achieve this objective, a therapeutic strategy must successfully navigate two distinct challenges: identifying the unique mutational landscape of an individual patient’s tumor and overcoming the profoundly immunosuppressive mechanics of the solid tumor microenvironment (TME). Currently, the field is focused on two dominant paradigms, both of which face significant biological and logistical bottlenecks. On one hand, personalized therapeutic cancer vaccines attempt to program the immune system ex ante but frequently encounter limitations due to antigen selection inaccuracies and inadequate adjuvant potency [1]. On the other hand, adoptive cellular immunotherapies, such as Chimeric Antigen Receptor (CAR) T-cell and Tumor-Infiltrating Lymphocyte (TIL) cell platforms, offer immediate cellular potency but often suffer from rapid exhaustion, poor trafficking, a lack of receptive surface solid tumor targets, acute toxicities, and prohibitive manufacturing constraints [2].

To address these combined constraints, this article outlines a translational framework designed to mitigate the logistical, temporal, and manufacturing limitations inherent to patient-specific autologous therapies while retaining the therapeutic benefits of a personalized anti-tumor response. The conceptual framework proposed herein, contextualized by recent Phase 2B clinical data [3], serves as an operational bridge that merges the economic and logistical advantages of off-the-shelf allogeneic cell biology with the clinical potential of personalized in situ antigen presentation. By utilizing the unmanipulated biology of living, pre-activated allogeneic Th1 cells as an active instructive agent, rather than a passive vehicle, this model is hypothesized to circumvent the requirement for ex vivo personalization across immunologically “cold” or treatment-refractory solid tumor phenotypes.

### 1.1. Conventional Cancer Vaccines

The clinical utility of neoantigen therapeutic cancer vaccines is constrained by a primary biological barrier: the identification of highly immunogenic tumor-specific antigens (TSAs) [4]. The majority of targetable proteins on solid malignancies are tumor-associated self-antigens (TAAs) [5]. Because these proteins are also expressed on healthy tissues, albeit at lower levels, the host’s endogenous T-cell repertoire is frequently depleted of high-affinity clones due to central and peripheral tolerance mechanisms [6,7]. Accordingly, attempting to vaccinate against TAAs often yields low-affinity immune responses and carries a persistent risk of off-target autoimmunity [8]. While next-generation sequencing (NGS) and bioinformatics have enabled the identification of patient-specific neoantigens arising from somatic mutations, predicting which neoantigens will effectively process and bind to a patient’s specific Human Leukocyte Antigen (HLA) molecules remains an ongoing challenge [9,10].

Another obstacle to designing an effective therapeutic cancer neoantigen vaccine is that tumors evolve dynamically to favor variant clones capable of evading host-mediated immune elimination [11]. Under selective immune pressure, this immunoediting process moves through elimination and equilibrium phases into an escape phase where resident cancer cells evade host immune control [12]. Consequently, a therapeutic vaccine intervention deployed during this escape phase directly encounters highly evolved, immunoedited tumor cell clones that have successfully bypassed host immune surveillance [13]. Immunotherapy protocols designed to enhance a resident anti-tumor immune response in late-stage disease may amplify an existing, non-effective immune response rather than generating a functional de novo cascade.

Crucially, historical clinical evidence from checkpoint and vaccine trials demonstrates that attempting to boost a compromised immune macroenvironment can correlate with hyperprogressive disease (HPD) [14], wherein an intervention may inadvertently accelerate tumor growth kinetics by expanding immunosuppressive regulatory T cells (Tregs) or triggering pro-tumorigenic inflammatory pathways. This clinical reality highlights the risk of amplifying a non-responsive immune loop and reinforces the utility of generating an entirely de novo immune architecture.

Compounding this antigen and immunoediting dilemma is a historical reliance on classical vaccinology frameworks designed to prevent infectious diseases by activating the humoral immune branch to elicit high titers of neutralizing antibodies [15]. While an antibody-centric approach is effective for neutralizing circulating pathogens, it has demonstrated limited efficacy in solid tumor oncology. Eradicating an established solid tumor requires a robust, immunologically “hot” cellular immune response—specifically the generation, trafficking, and infiltration of cytolytic CD8+ T cells and type 1 helper (Th1) T cells [16]. Traditional cancer vaccines frequently lack the adjuvant potency to induce this intense cellular cascade [17]. When a synthetic peptide or mRNA formulation is administered without a powerful, localized inflammatory context, it typically fails to break systemic immune tolerance, leading to the exclusion or rapid deactivation of weakly primed host T cells by the unadjuvanted tumor stroma [18].

### 1.2. Adoptive Cellular Immunotherapies

While therapeutic vaccines aim to generate an endogenous immune response ex ante, adoptive cellular immunotherapies attempt to bypass poor host priming by directly infusing large numbers of functional effector cells. This paradigm has achieved historic, curative successes in hematological malignancies—particularly with Chimeric Antigen Receptor (CAR) T-cell platforms targeting lineage-specific antigens in leukemias and lymphomas. Furthermore, adoptive cellular platforms have achieved major clinical breakthroughs in highly mutated solid tissue types, exemplified by the recent regulatory approvals of expanded autologous Tumor-Infiltrating Lymphocyte (TIL) regimens for advanced, refractory metastatic melanoma. Despite these foundational milestones, translating these clinical successes across the wider landscape of poorly immunogenic, classically “cold” epithelial solid malignancies has revealed distinct biological and structural boundaries.

To substantiate these boundaries, published human clinical datasets confirm that conventional autologous cell manufacturing remains heavily constrained by a multi-week operational lag and a high risk of final product failure due to the phenotypic exhaustion of host cells harvested from heavily pretreated cancer patients [2,19]. In contrast, clinical evaluations of the non-engineered, off-the-shelf allogeneic Th1 framework demonstrate immediate point-of-care availability, completely bypassing these logistical manufacturing constraints while driving robust, predictable systemic immune activation in late-stage oncological cohorts without ex vivo waiting periods [3,20]. While exploratory clinical evaluations of the non-engineered, off-the-shelf allogeneic Th1 framework suggest immediate point-of-care availability without ex vivo manufacturing delays, substantial uncertainties remain regarding the absolute predictability of this approach. Because the platform acts indirectly as an upstream instructor, its clinical efficacy is entirely dependent on the variable, unverified baseline immune competence of a heavily pretreated host. If a patient harbors absolute lymphoid or myeloid depletion, the therapeutic capacity to initiate systemic activation will be fundamentally compromised, representing a major clinical uncertainty that requires extensive validation against the immediate effector potency of autologous platforms.

#### 1.2.1. Target Recognition and Lineage Depletion Constraints in Solid Tumors

A critical vulnerability of CAR-engineered cells is their reliance on synthetic biology to force an immune cell to recognize a malignancy via an inserted immunoglobulin (Ig)-derived molecule, specifically an antibody-based single-chain variable fragment (scFv) [21]. Consequently, this design paradigm is subject to the surface-target limitations that restrict humoral immunity, confining cellular recognition strictly to intact, surface-expressed proteins. This restriction creates an acute target bottleneck in solid tumor oncology, where the vast majority of true tumor-specific neoantigens reside as intracellular oncogenic proteins. Native cytotoxic T lymphocytes (CTLs) evolved to recognize these hidden targets by scanning internally processed peptide fragments presented on surface Major Histocompatibility Complex (MHC) molecules [22]. By operating through an antibody-like surface interaction rather than a native, MHC-restricted T-cell receptor (TCR) pathway, CAR-T cells typically remain incapable of targeting intracellular mutations, rendering them vulnerable to tumor antigen escape via surface target downregulation [23].

Crucially, engineering a T cell to recognize antigens through an antibody-like surface mechanism could explain why this paradigm functions effectively in hematological malignancies yet presents profound translational barriers in solid tumors. In blood cancers, the clinical objective is typically the wholesale depletion of an entire cell lineage, such as CD19+ or CD20+ B cells. Because the human body can tolerate the temporary or permanent depletion of a non-essential cell class, managed clinically with intravenous immunoglobulin (IVIG) replacement therapy, an antibody-derived CAR can systematically eliminate antigen-positive cells across both healthy and malignant compartments. This broad-spectrum targeting approach does not easily translate to solid tumors, which are derived from essential normal tissues. Solid malignancies rarely express truly unique surface proteins; instead, they display tumor-associated antigens (TAAs) that are extensively shared with healthy, vital epithelial organs [24,25]. Because an antibody-style CAR cannot easily differentiate between low-level baseline expression on a normal epithelial cell and high-level overexpression on a carcinoma cell, attempting to apply this lineage-depletion framework to solid tumor oncology presents a persistent risk of severe “on-target, off-tumor” toxicities [26].

#### 1.2.2. Systemic Toxicities and Genotoxic Risks in Engineered Platforms

The synthetic potency and autonomous expansion of CAR-T cells can induce severe acute toxicities, most notably Cytokine Release Syndrome (CRS) and Immune Effector Cell-Associated Neurotoxicity Syndrome (ICANS) [27,28]. Managing these hyper-inflammatory conditions frequently requires intensive care monitoring and aggressive countermeasures, including anti-interleukin 6 (IL-6) monoclonal antibodies or high-dose corticosteroids, which may inadvertently compromise final anti-tumor efficacy. Paralleling these CAR-T liabilities are the distinct clinical toxicities associated with TIL therapy frameworks [29]. Because TIL platforms rely on expanding exhausted autologous cells, successful clinical deployment currently necessitates high-dose pre-conditioning chemotherapy to induce host lymphodepletion, followed by sequential high-dose interleukin-2 (IL-2) infusions to promote in vivo cell survival and expansion. This intensive ancillary regimen causes prolonged cytopenias, opportunistic infections, and capillary leak syndrome characterized by hypotension, generalized anasarca, or transient pulmonary and renal impairment [19].

Over the long term, a reliance on viral vectors or advanced gene-editing tools introduces additional risks regarding genomic instability. Insertional mutagenesis can disrupt host tumor suppressor genes or activate oncogenes—a clinical reality highlighted by recent regulatory investigations and subsequent mandated boxed warnings regarding cases of secondary T-cell malignancies arising following engineered cellular interventions [30,31]. Collectively, these acute, systemic, and genotoxic risks demonstrate that current engineered cellular platforms impose significant physiological demands on heavily pretreated cancer patients, reinforcing the utility of exploring non-lymphodepleted, self-limiting cellular architectures.

#### 1.2.3. Synthetic Engineering Strategies and Alternative Platforms in Adoptive Cell Therapy

In response to these multi-faceted biological barriers, the adoptive cell therapy field has engaged in increasingly complex synthetic biological engineering to introduce artificial functionality into therapeutic cell lines [32]. Pharmacological “off-switches” and reversible control circuits utilize inducible suicide genes or small-molecule-disrupted CAR binding domains to mitigate runaway toxicities [33], though they require continuous external drug monitoring and add significant complexity to manufacturing processes [34]. Simultaneously, complex hypo-immune editing strategies use CRISPR/Cas9 to disrupt endogenous T-cell receptors and surface MHC molecules to evade host rejection [35,36]; however, this can inadvertently trigger “missing-self” destruction by host natural killer (NK) cells and frequently requires further transgene overexpression of protective ligands like HLA-E or CD47 [37]. Researchers are also engineering “armored” CAR T-cells designed to modulate the tumor stroma by transfecting them to secrete anti-PD-1 or anti-PD-L1 antibodies alongside homeostatic cytokines [38,39], which can similarly elevate insertional mutagenesis risks.

To mitigate T-cell-specific toxicities and avoid GvHD risks, the field has investigated the engineering of allogeneic Chimeric Antigen Receptor Natural Killer (CAR-NK) cells as an off-the-shelf alternative [37]. While CAR-NK platforms often offer a more favorable acute safety profile due to a reduced propensity for runaway cytokine release, they typically exhibit limited in vivo persistence, restricted trafficking into dense solid masses, and rapid functional suppression within the suppressive TME [40]. Crucially, by overlaying an antibody-derived CAR construct onto the NK cell, this engineering strategy confines the cell to the same surface-restricted, MHC-blind recognition paradigm that limits conventional CAR-T cells. This synthetic constraint may underutilize the NK cell’s native evolutionary ability to employ a diverse array of germline-encoded receptors to scan for broad, intracellular metabolic stress signals, missing-self ligands, and MHC class I down-regulation across heterogeneous tumor populations.

Attempting to bypass the logistical friction and high costs of ex vivo cell manufacturing, recent research has pivoted toward in vivo CAR-T cell generation utilizing targeted viral vectors, engineered lipid nanoparticles (LNPs), or modified mRNA formulations administered directly to genetically reprogram endogenous host T cells in situ [41]. Underlying genetic material delivery directly into the systemic circulation carries a risk of off-target transduction, potentially altering non-T cell populations or healthy tissues, such as hepatocytes [42]. Furthermore, in vivo viral integration reintroduces permanent genotoxic risks and insertional mutagenesis within the host genome. Alternatively, transient mRNA-LNP approaches frequently suffer from insufficient or short-lived CAR expression, failing to sustain the durable effector response needed to eradicate dense solid malignancies [43]. Crucially, even when successfully generated in vivo, these cells remain restricted by their antibody-derived scFv architecture, leaving them structurally blind to internal oncogenic mutations presented on host MHC molecules and vulnerable to surface antigen escape.

This operational limitation establishes a distinct mechanistic dichotomy between existing universal effector cell strategies and the alternative theoretical framework evaluated in this Perspective. While conventional off-the-shelf platforms view host rejection as a clearance liability, the proposed strategy shifts the focus by utilizing the living allogeneic cell not as a direct cytolytic executioner but as a temporary biological instructor. Instead of striving to prolong the survival of an adoptive allogeneic line, this model intentionally prompts a controlled host-versus-graft (HvG) rejection cascade to serve as a macroenvironmental catalyst. By forcing this transient tissue clearance to take place within a defined sequence of dermal priming and vascular rechallenge, the framework exploits the host’s innate anti-allo rejection machinery to break systemic tolerance and remodel the suppressive stroma. Consequently, rather than delivering a short-lived adoptive effector function that vanishes upon rejection, the non-engineered allogeneic input serves strictly to orchestrate an endogenous, host-mediated, polyclonal anti-tumor response. This paradigm shift is conceptualized to allow a patient-specific adaptive immune response to develop within the host’s memory architecture; however, whether this indirect host-mediated response can reliably generate sufficient high-affinity clones to match the direct, immediate cytolytic density and targeted tumor-killing velocity delivered by gene-engineered, armored effector platforms remains a primary biological question.

### 1.3. Mechanical Reorientation: Comparing Synthetic Modification and Native Biological Frameworks

While synthetic engineering strategies represent notable advancements in biotechnology [44,45], they introduce complex, artificial biological layers to navigate obstacles that might otherwise be managed through native cellular interactions. Each additional transgene, synthetic logic gate, or knock-out circuit can amplify manufacturing costs, increase the risk of insertional mutagenesis, compromise long-term T-cell fitness, and elevate regulatory hurdles for clinical translation.

The theoretical framework described in this article provides an alternative approach to these engineered designs. Instead of introducing genetic modifications to allow allogeneic cells to evade host detection, persist long-term, or deliver synthetic payloads, this model shifts the therapeutic mechanism toward unmanipulated cellular interactions. By employing the native biology of living, allogeneic type 1 helper (Th1) cells, the framework utilizes the host’s endogenous immune pathways that mediate the clearance of foreign tissue. Rather than attempting to suppress this host-versus-graft (HvG) interaction, the model is designed to harness an immediate, high-potency immune clearance response as an active immunomodulatory mechanism capable of driving endogenous immunity.

To evaluate the mechanical feasibility of this approach, the subsequent discussion outlines the multi-phase operational biology undergirding the model. This framework details how initial, localized intradermal priming is proposed to expand a host anti-alloantigen Th1 memory pool, and how a subsequent systemic intravenous rechallenge may trigger a controlled, Type 1 cytokine-driven cascade. Mechanistically, this systemic interaction is hypothesized to non-specifically activate circulating host memory cells and natural killer (NK) cells, promoting their extravasation and trafficking into metastatic tumor sites to convert immunologically “cold” tumors to inflamed phenotypes.

The analysis further examines how this downstream cellular cascade could remodel the immunosuppressive stroma of cold tumors and induce innate, NK-mediated immunogenic cell death (ICD) at malignant sites. Ultimately, this article outlines how the strategic coupling of allogeneic priming and systemic rejection could facilitate substantial modification of the TME and localized ICD, satisfying the physiological requirements for effective in situ vaccination. By converting the established tumor mass into an endogenous antigen source, this multi-wave process is hypothesized to stimulate the host’s native immune system to capture internally processed peptides and mount a personalized, patient-specific anti-tumor adaptive immune response—achieved through a non-genetically manipulated therapeutic cell line in immunologically “cold” or treatment-refractory solid tumor phenotypes.

### 1.4. Comparative Paradigms of Non-Engineered, Off-the-Shelf Allogeneic Cell Platforms

While the proposed theoretical framework utilizes non-engineered, fully mismatched allogeneic Th1 cells explicitly to trigger a controlled HvG rejection cascade, a parallel domain of allogeneic cellular immunotherapy explores the deployment of naturally occurring, universal donor T-cell platforms [46]. These platforms seek to achieve direct, off-the-shelf therapeutic targeting without the requirement for complex, multiplexed gene editing to disrupt endogenous alpha-beta (α/β) T-cell receptors (TCRs) or Human Leukocyte Antigen (HLA) complexes. Broadly, these non-engineered universal strategies can be biophysically segregated into two distinct operational paradigms: conventional HLA-restricted donor banking targeted to conserved shared antigens, and invariant, HLA-independent lineages that recognize non-peptide metabolites, lipids, or phosphoantigens [47,48]. A foundational characteristic shared by these universal donor concepts is their operational reliance on the transferred allogeneic cell to serve as the primary, long-term cytotoxic effector. Consequently, because these therapies treat host rejection as a clearance liability, the eventual elimination of the donor cells by the recipient’s immune system restricts sustained anti-tumor efficacy (See Table 1 for comparison of the proposed framework and other paradigms).

The first of these effector-focused paradigms relies on the systematic selection and banking of conventional α/β cytotoxic T lymphocytes (CTLs) derived from healthy, virus-seropositive allogeneic donors. This approach utilizes the natural biology of virus-specific T cells (VSTs)—predominantly those directed against Epstein–Barr Virus (EBV), Cytomegalovirus (CMV), or Adenovirus—to recognize well-defined, HLA-restricted viral or tumor-associated targets [49,50]. Because these cells possess endogenous TCRs focused on viral peptide-MHC complexes, they exhibit a restricted capacity to react against mismatched host normal tissues, which minimizes the clinical risk of acute Graft-versus-Host Disease (GvHD).

To deploy these cells off-the-shelf against malignancies, matching algorithms pair a patient’s specific HLA allele restriction with a corresponding pre-manufactured donor cell batch from a centralized master bank. This enables the direct targeting of shared public neoantigens or highly conserved viral oncogene fragments. However, the therapeutic window of this conventional platform remains constrained by the requirement for partial HLA matching. In patients presenting with complete HLA mismatching or tumor-induced locus downregulation, these cells face rapid host-mediated clearance or target escape, potentially resulting in functional termination before adequate tumor debulking occurs [51].

To circumvent the biological boundaries imposed by HLA polymorphism and subsequent target loss, research focuses on invariant, HLA-independent T-cell lineages that exploit conserved, non-polymorphic presentation machinery to recognize non-peptide antigens across broad patient populations without triggering GvHD. Within this landscape, gamma-delta (γδ) T cells, predominantly the γ9/δ2 T-cell subset [52], bypass standard MHC class I and II restriction to directly recognize intracellular phosphoantigens and metabolic intermediates, such as isopentenyl pyrophosphate (IPP), which accumulate abnormally within malignant cells due to dysregulated mevalonate pathway kinetics [53].

Concurrently, mucosal-associated invariant T (MAIT) cells represent an innate-like T-cell population characterized by a semi-invariant αβTCR structure that recognizes small-molecule riboflavin metabolites and microbial precursors presented in the context of the monomorphic, universally conserved MHC class I-related protein 1 (MR1) [54]. Because MR1 is invariant across the human population, allogeneic MAIT cells can be transferred into unmatched hosts without inducing alloreactive tissue destruction. This non-polymorphic targeting paradigm is closely mirrored by invariant natural killer T (iNKT) cells, which express a restricted TCR α/β-chain interfacing specifically with CD1d, a monomorphic MHC class I-like molecule that presents endogenous and exogenous lipid antigens, such as alpha-galactosylceramide (αGalCer), to secrete waves of Th1-polarized cytokines upon ligation [55].

While these naturally universal cellular chassis reduce the clinical threat of iatrogenic GvHD without synthetic genetic modifications, their therapeutic efficacy within dense solid malignancies remains restricted by distinct physiological barriers. Because these cells do not purposefully manipulate HvG rejection kinetics, they remain vulnerable to host-mediated immunological clearance. Upon intravenous infusion into an immunocompetent host, the recipient’s resident NK cells and alloreactive CTLs can rapidly clear these non-engineered lines via “missing-self” or direct allorecognition cascades within a compressed window [56].

Furthermore, these platforms rely on the assumption that universal donor cells can autonomously traffic into, survive within, and dismantle the immunosuppressive solid tumor microenvironment (TME). Without active manipulation of the host’s systemic macroenvironment or localized endothelial integrin activation loops, these universal lineages frequently encounter physical exclusion at the desmoplastic border or rapid metabolic exhaustion within the adenosine-rich, hypoxic tumor stroma [57]. Consequently, while these platforms present an elegant biological solution to prevent toxic GvHD, their operational lifespan as isolated monotherapies is frequently brief, underscoring the potential translational requirement for platforms that strategically align host-donor incompatibility as an active instructive catalyst rather than a passive target.

This operational sequencing establishes a distinct mechanistic dichotomy between conventional platforms and the current framework. While synthetic cell lines view host rejection as a clearance liability, this strategy uses it as an active immunomodulatory asset. The fundamental novelty resides in three interconnected parameters: (a) Mechanistic Sequencing: Moving from an isolated dermal priming phase to a systemic vascular rechallenge; (b) Therapeutic Intent: Shifting the primary cytolytic burden away from the infused donor cells and permanently transferring it back to a newly cross-primed endogenous host immune repertoire; and (c) Biological Leverage: Exploiting the high evolutionary potency of host-versus-graft (HvG) mismatch responses to induce uniform endothelial integrin activation, bypassing the localized physical exclusion barriers that typically restrict standard in situ vaccination modalities. Crucially, however, the long-term functional durability, clonal expansion limits, and potential exhaustion kinetics of this newly primed host-derived repertoire face considerable physiological uncertainties compared to continuously active, gene-armored or multi-antigen universal platforms, representing a vital boundary that remains to be explored in randomized clinical trials.

Instead of attempting to prolong the survival of an adoptive allogeneic line, this model intentionally prompts a controlled HvG) rejection cascade to serve as a macroenvironmental catalyst. By forcing this transient tissue clearance to take place within a defined sequence of dermal priming and vascular rechallenge, the framework exploits the host’s innate anti-allo rejection machinery to break systemic tolerance and remodel the suppressive stroma. Consequently, rather than delivering a short-lived adoptive effector function that vanishes upon rejection, the non-engineered allogeneic input serves strictly to orchestrate an endogenous, host-mediated, polyclonal anti-tumor response. This paradigm ensures that even after the donor cells have been cleared by the host, a durable, patient-specific adaptive immune response remains established within the patient’s own newly generated memory architecture.

**Table 1:** Operational Matrix: Effector-Focused Universal Donor Platforms vs. Proposed Instructive Strategy.

Parameter	Effector-Focused Platforms (VSTs, γδ T, MAIT, iNKT)	Proposed Instructive Framework (Allogeneic Th1)
Primary Cell Source	Healthy donor allogeneic subsets or cell lines [46].	Purified, non-engineered healthy donor CD4+ Th1 cells.
Target Recognition Mechanism	Surface-restricted, MHC-restricted, or monomorphic ligand scanning [49].	Unrestricted; acts upstream as a biological instructor via host APC licensing.
Host Rejection (HvG) Relationship	Treated as a clearance failure mode and a primary survival liability [56].	Intentionally prompted as an active macroenvironmental catalyst.
Primary Cytolytic burden	Borne directly by the infused donor cells [48].	Permanently transferred back to the freshly cross-primed host immune system.
Therapeutic Target Scope	Confined to shared public antigens or invariant ligands [54].	Patient-specific, highly polyclonal private mutation-associated neoantigens (MANA).

### 1.5. Theoretical Mechanisms of Immunoediting Reversal and In Situ Vaccination

The baseline presence of macroscopic clinical disease in late-stage oncological cohorts provides evidence of the failure of the initial host immunosurveillance phase, while advanced progression highlights a subsequent collapse of the immune-mediated tumor equilibrium phase of immunoediting. Therefore, to design an effective therapeutic approach for patients in the escape phase, an immunotherapeutic protocol must be conceptualized to reverse or bypass the immunoediting process. This reorientation requires presenting Mutated Associated Neoantigens (MANA)—expressed within the escaped resident lesions—to the host immune system within an inflammatory context modeled to generate a functional, de novo anti-tumor response against immune-edited variant clones. Subsequently, this de novo cellular response must modulate the existing, suppressed host immune macroenvironment, seeking to mitigate the systemic Type 2 (Th2) polarization and regulatory T-cell (Treg) biases that can cause traditional immune-boosting platforms to correlate with hyperprogressive disease (HPD) [58,59].

An effective strategy for priming naïve T cells in late-stage disease necessitates the re-presentation of MANA within a TME modulated to express high levels of inflammatory cytokines, co-stimulatory molecules, and endogenous danger signals [60]. The re-introduction of tumor antigens under these active inflammatory conditions is hypothesized to generate the precise microenvironmental conditions necessary to achieve an in situ vaccine (ISV) effect [61,62]. This approach offers distinct mechanistic features over traditional personalized vaccine platforms, targeted protein degraders (PROTACs) [63], or isolated pattern recognition receptor (PRR) and STING agonists [64]: it is conceptualized to prime a patient-specific, de novo anti-tumor cellular immune response in vivo, potentially resulting in an adaptive repertoire customized to the patient’s evolving tumor architecture without the operational or manufacturing constraints associated with pre-identifying, sequencing, and isolating individual patient-specific neoantigens ex ante [65].

Clinical precedent for the utility of such an in situ vaccination framework exists within the documented phenomenon known as the abscopal effect [66,67]. Classically observed following local palliative radiation therapy, the abscopal effect refers to the spontaneous regression of distant, non-irradiated metastatic lesions. Mechanistically, local radiotherapy acts as an external catalyst for tumor destruction, occasionally forcing the localized tumor mass to serve as a functional site of in situ vaccination. However, the rarity of the abscopal effect in clinical practice highlights a limitation of radiation oncology: radiotherapy predominantly induces non-immunogenic, apoptotic cell death rather than robust immunogenic cell death (ICD) [68]. Apoptosis is inherently a silent, tolerogenic clearance process that frequently lacks the necessary danger signals to break systemic immune tolerance.

Furthermore, for an in situ vaccine effect to successfully drive systemic adaptive immunity, the release of tumor neoantigens must occur within an actively inflamed, co-stimulatory microenvironment [69]. In the majority of metastatic settings, the unmodulated tumor stroma remains immunosuppressive and deficient in the inflammatory cytokines [70] needed to mature arriving antigen-presenting cells (APCs) [71]. Radiotherapy achieves the abscopal effect inconsistently because it cannot systematically remodel this hostile background. Nevertheless, it establishes a critical clinical principle: forcing systemic antigen exposure through localized tumor destruction can drive a systemic, polyclonal adaptive immune response capable of targeting micro-metastases, provided the required immunogenic and inflammatory criteria are satisfied.

The execution of a managed ISV protocol relies on replicating this phenomenon systematically by forcing the deliberate, localized release of MANA into a modified tumor stroma for subsequent capture and processing by host APCs. Intracellularly, these somatic mutations are frequently found complexed with chaperoning heat shock proteins (HSPs) such as gp96, HSP70, and HSP90 [72,73]. Forced release of internal MANA in situ requires tumor cells to undergo ICD, which is characterized by the coordinated translocation and release of damage-associated molecular patterns (DAMPs) into the TME, including uric acid, extracellular ATP [74], and High-Mobility Group Box 1 (HMGB1) protein [75]. These DAMPs are recognized by pattern recognition receptors (PRRs), such as Toll-like receptors (TLRs), expressed on local host APCs. The resulting DAMP-TLR activation of APCs facilitates the development of adaptive anti-tumor immune responses specific for the neoepitopes presented alongside MHC Class I and Class II molecules [76].

To induce downstream ICD within this clinical protocol, a critical mechanistic step involves the activation of circulating host natural killer (NK) cells. Activated NK cells are poised to eliminate target tumor cells via the exocytosis of lytic granules containing perforin and granzyme, mechanically disrupting the tumor cell membrane [77]. Concurrently, this cytolytic interaction forces the surface exposure of calreticulin and the release of chaperoned HSP-MANA complexes alongside corresponding DAMP signals. Simultaneously, these activated NK cells secrete pro-inflammatory cytokines such as IFN-γ and TNF-α to modulate the local TME, repolarizing suppressive myeloid subsets and facilitating dendritic cell cross-presentation [78,79]. To systematically orchestrate this multi-wave cellular event without synthetic engineering or reliance on the variable mechanics of radiotherapy, a multi-phase operational model utilizing unmanipulated, pre-activated allogeneic Th1 cells is evaluated.

## 2. The Multi-Phase Operational Model

### 2.1. Overview of the Allogeneic Instructive Cascade

To systematically address the clonal immunoediting escape phase and achieve a durable in situ vaccination effect using non-genetically modified, allogeneic activated type 1 helper (Th1) cells, the proposed therapeutic framework coordinates a series of sequential immunological transformations. Rather than approaching the tumor stroma as a static target, this operational model leverages the dynamic kinetics of a host-versus-graft (HvG) rejection cascade to modulate the host immune environment [80].

**Figure 1:**
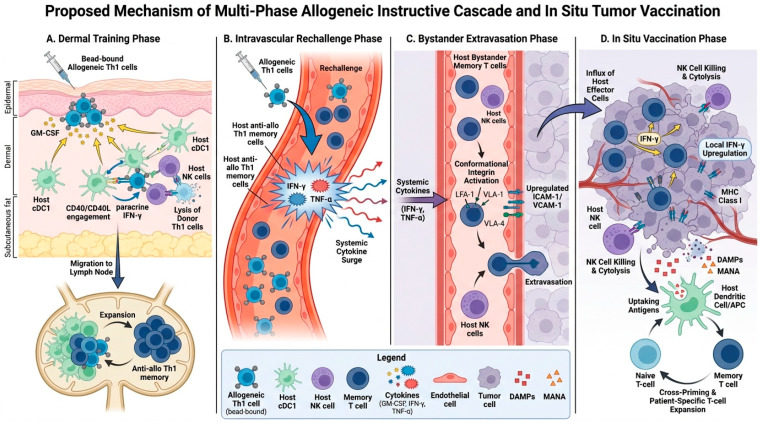
**Proposed Mechanism of the Multi-Phase Allogeneic Instructive Cascade and In Situ Tumor Vaccination**. This schematic outlines a hypothesized immunological workflow designed to convert an immunologically cold tumor into an inflamed zone using an off-the-shelf cell catalyst. Note: This cascade outlines a proposed theoretical mechanism that has not yet been clinically or experimentally validated. (**A**) Dermal Training Phase: Intradermal injection of bead-bound, mismatched allogeneic Th1 cells establishes a Type 1 microenvironment. Local GM-CSF secretion recruits host conventional type 1 dendritic cells (cDC1s), which are licensed into mature IL-12+ phenotypes via CD40L-CD40 engagement and paracrine IFN-γ signals while host NK cells execute donor cell lysis. Licensed host cDC1s process the liberated alloantigens and migrate to regional lymph nodes to expand a systemic host anti-allo Th1 memory pool. (**B**) Intravascular Rechallenge Phase: Subsequent intravenous infusion of donor Th1 cells triggers a rapid encounter with circulating host anti-allo memory cells, driving a sharp, self-limiting systemic wave of IFN-γ and TNF-α. (**C**) Bystander Extravasation Phase: The systemic cytokine surge activates the tumor endothelial wall, upregulating cell-adhesion molecules (ICAM-1 and VCAM-1) to induce conformational integrin activation (LFA-1 and VLA-4) on circulating host bystander memory T cells and NK cells, directing their diapedesis into the tumor stroma. (**D**) In Situ Vaccination Phase: Infiltrating host effector populations remodel the suppressive stroma. Localized IFN-γ upregulates tumor MHC Class I expression, while extravasated host NK cells execute cytolysis, inducing immunogenic cell death (ICD). This releases soluble damage-associated molecular patterns (DAMPs) and private mutation-associated neoantigens (MANA), allowing host antigen-presenting cells to cross-prime a de novo, patient-specific adaptive anti-tumor T-cell repertoire.

This orchestrated process is modeled to unfold through five sequential, interdependent immunological phases (see Figure 1), initiating with a deliberate modulation of the host’s systemic helper T-cell balance to shift the macroenvironment away from a progressive, tumor-promoting Type 2 (Th2) bias. By promoting homeostatic Type 1 (Th1) differentiation, this foundational step is designed to generate an expanded pool of functional, allo-specific Th1 memory cells within the peripheral circulation. Subsequently, the framework seeks to convert characteristically “cold,” immunologically excluded, or microsatellite stable (MSS/pMMR) solid tumor lesions into inflamed phenotypes. This transformation is driven by the broad, non-specific activation of circulating host allo-specific Th1 memory cells and natural killer (NK) cells via a bystander effect [81,82] resulting from the cytokine release upon host rejection of an intravenous (IV) infusion of living, activated allogeneic Th1 cells, promoting their extravasation and trafficking across structural stromal barriers into metastatic lesions.

Once these activated host memory Th1 cells and NK cell populations have infiltrated the tumor stroma, the proposed protocol is conceptualized to satisfy the physiological criteria necessary to induce an in situ vaccine (ISV) effect and drive de novo neoantigen priming [61,62]. This is modeled to occur by inducing host NK-mediated immunogenic cell death (ICD) within a modified, inflammatory context characterized by localized IFN-γ signaling. To prevent newly recruited host effector populations from undergoing rapid deactivation, the cascade is hypothesized to target local tumor immunoavoidance pathways by sustaining a continuous, localized inflammatory cytokine loop rich in Interleukin-12 (IL-12) and IFN-γ. This localized inflammation is hypothesized to suppress regulatory myeloid and T-cell subsets, neutralizing the tumor’s protective stromal shield [64]. Ultimately, the protocol seeks to drive the systemic expansion and long-term secondary immunoenhancement of the freshly primed, patient-specific anti-tumor adaptive immune response, creating a durable memory response capable of patrolling the macroenvironment to mitigate future metastatic recurrence [65].

The operational execution of this multi-phase cascade depends on using the living allogeneic Th1 cells not as a direct cytolytic executioner but as a dynamic biological instructor. By separating the therapeutic intervention into distinct anatomical stages—moving from localized dermal training to systemic vascular rechallenge—the framework exploits the host’s innate anti-allo rejection machinery to break systemic tolerance. This approach represents an alternative framework in cellular immunotherapy; rather than deploying expensive, engineered autologous cells that can be prone to T-cell exhaustion and restricted by surface antigen target limitations [23,26], this biology-driven model relies on the native programming of host effector networks. This process is designed to utilize an off-the-shelf, non-engineered allogeneic cell formulation to facilitate a personalized adaptive immune response in vivo.

### 2.2. Intradermal Priming and Cellular Mechanics of Dermal Allorecognition

The operational execution of this instructive cascade begins within the cutaneous basement membrane—an anatomical compartment selected for its dense network of professional antigen-presenting cells (APCs), including Langerhans cells (LCs) and conventional dendritic cells (DCs) [57]. Iterative intradermal (ID) injections of living, pre-activated allogeneic Th1 cells are administered to establish a localized, immunogenic microenvironment. Because these donor cells express mismatched, non-self, human leukocyte antigens (HLAs) alongside a high density of functional surface CD40L and activation ligands, they trigger immediate recognition by the host’s dermal sentinel network. This local mismatch interaction initiates a controlled host-versus-graft (HvG) rejection process that operates simultaneously via both direct host recognition of foreign donor major histocompatibility complex (MHC) molecules and the indirect processing and presentation of donor cellular fragments by host dermal APCs, such as LCs [83,84,85].

Crucially, because the allogeneic cellular formulation maintains an active, unmanipulated Th1 phenotype, it secretes an array of pro-inflammatory cytokines, including interferon-gamma (IFN-γ), tumor necrosis factor-alpha (TNF-α), and granulocyte-macrophage colony-stimulating factor (GM-CSF). Mechanistically, the localized secretion of GM-CSF serves as a chemokine gradient, recruiting host immature DCs directly into the site of the allogeneic interaction. Host APCs engulfing this allogeneic material after rejection are subsequently conditioned within an environment featuring Type 1 inflammatory signals. This dermal rejection zone serves as an active priming site for the host’s adaptive immune system.

The simultaneous interaction with high-density donor-expressed CD40L and donor-secreted exogenous IFN-γ is hypothesized to satisfy the precise, two-signal maturation requirement necessary to license and mature host Type 1 dendritic cells (DC1s). This distinct molecular conditioning is conceptualized to drive host APC maturation, upregulate CD80, CD86, and CD40 co-stimulatory surface markers, and promote the sustained secretion of endogenous Interleukin-12 (IL-12). Once transitioned into this active IL-12+ DC1 phenotype, these host cells migrate to the draining lymph nodes to prime and expand host-derived, naive T-cell pools, driving their differentiation down a Type 1 pathway and expanding allo-specific helper and cytotoxic T lymphocyte (CTL) populations.

Preclinical evaluations in Th2-biased murine models and healthy human cohorts demonstrate that repeated, scheduled intradermal allogeneic exposure generates a durable pool of circulating allo-specific memory Th1 cells [20,86,87]. Significantly, this Th1 adjuvant effect occurs when the injected allogeneic Th1 cells are in an activated state, and this operational framework has been reported to elicit tumor-specific immunity in prior investigated models [86,88]. Through these repeated intradermal boosting cycles, the protocol is hypothesized to expand the frequency of circulating host anti-allo T-cell populations, shifting the systemic Th1/Th2 balance and establishing the necessary systemic cellular baseline prior to vascular intervention.

### 2.3. Systemic Rechallenge and Kinetics of the Intravascular Allogeneic Response

Following systemic allogeneic priming in the dermal compartment, the therapeutic protocol transitions to an intravenous (IV) cell administration strategy. This subsequent vascular stage is theoretically conceptualized to stimulate the expanded, circulating host allo-specific memory Th1 pool and innate effector cells to extravasate into metastatic lesions, aiming to convert uninflamed or immunologically excluded solid tumor tissues into inflamed, cell-dense sites. The IV infusion of the living allogeneic Th1 cell formulation into an allo-primed host initiates a rapid, systemic intravascular rejection cascade. Because the host macroenvironment possesses an expanded pool of anti-allo memory cells, this immediate host-versus-graft mismatch reaction stimulates a pronounced upregulation of circulating IFN-γ and tumor necrosis factor-alpha (TNF-α) levels [89,90].

Crucially, while this intravascular rejection event drives a potent systemic cytokine release, it is mechanistically distinct from, and does not trigger, the severe clinical toxicity profile associated with Cytokine Release Syndrome (CRS) [91]. This safety profile is governed by a divergence in cellular and molecular kinetics. Clinical CRS is primarily a monokine-driven phenomenon, wherein the continuous, autonomous expansion of engineered cell therapies hyper-activates host monocytes and macrophages, triggering an escalating feedback loop of pyrogenic monokines, specifically Interleukin-1 (IL-1) and Interleukin-6 (IL-6) [92,93]. In contrast, the systemic response induced under this framework represents a controlled, self-limiting T-cell cytokine release profile. Because these non-genetically manipulated allogeneic Th1 cells are incapable of autonomous in vivo persistence and are rapidly cleared by the host immune system within a predictable physiological window, they do not sustain the macrophage over-activation loops necessary to drive severe monokine-mediated toxicity.

Instead, this transient T-cell cytokine release mediates broad anti-tumor immune activity and induces “bystander activation” of both adaptive memory T cells and host natural killer (NK) cells [94]. This cytokine-driven activation bypasses the strict requirement for direct T-cell receptor (TCR) antigen engagement, driving the upregulation of effector markers and licensing host NK cells to exert innate cytotoxic activity capable of targeting Major Histocompatibility Complex (MHC)-deficient or immunoedited tumor subclones that frequently evade traditional adaptive surveillance [95,96].

This systemic and peri-tumoral exposure to elevated IFN-γ and inflammatory cytokine gradients is hypothesized to modulate the functional tracking and homing phenotypes of circulating lymphocytes [97]. Mechanistically, the intravascular cytokine release upregulates critical integrin complexes—specifically lymphocyte function-associated antigen-1 (LFA-1; CD11a/CD18) and very late antigen-4 (VLA-4; α4β1)—on the surfaces of circulating bystander-activated host Th1 and NK cell populations. Concurrently, systemic IFN-γ signals stimulate the tumor vascular endothelium to overexpress corresponding cell-adhesion molecules, including Intercellular Adhesion Molecule-1 (ICAM-1) and Vascular Cell Adhesion Molecule-1 (VCAM-1). This synchronized receptor-ligand upregulation reduces the forward velocity of circulating host lymphocytes along the tumor endothelial walls, initiating the classical cascade of rolling, tethering, firm adhesion, and transendothelial extravasation into malignant tissue beds [98].

Once localized within the previously privileged tumor microenvironment (TME), these infiltrating, activated host cells release concentrated waves of IFN-γ and Type 1 cytokines. This localized secretion alters the transcriptional profile of host stromal components, endothelial cells, and malignant cells, stimulating them to synthesize and secrete the downstream C-X-C motif chemokines CXCL9, CXCL10, and CXCL11 [99]. These inflammatory chemokines function as a localized chemotactic gradient, binding to surface CXCR3 and CCR5 receptors on un-activated circulating host cells to recruit additional conventional dendritic cells and antigen-experienced lymphocytes directly into the TME [100]. With each subsequent iterative intradermal priming and intravenous rechallenge cycle, this cellular influx is enhanced. The proposed framework seeks to establish a self-amplifying positive feedback loop driven by Th1-derived IFN-γ and host APC-derived Interleukin-12 (IL-12) that modulates local immunosuppressive stasis, disrupts myeloid-derived suppressor cell (MDSC) exclusion networks, and modifies the structural architecture of the tumor stroma for robust tumor lysis [101].

### 2.4. In Situ Vaccination, Host NK-Mediated Lysis, and Adaptive Cross-Priming

The primary objective of converting the tumor microenvironment (TME) from immunologically “cold” to inflamed is to establish the physiological conditions required to drive tumor immunogenic cell death (ICD) within an active inflammatory context, facilitating the subsequent assembly of an in situ vaccine (ISV). While conventional cytotoxic modalities such as ionizing radiation can induce standard forms of tumor necrosis, the resulting release of unchaperoned tumor antigens frequently fails to generate systemic anti-tumor responses—classically termed the abscopal effect—due to the background immunosuppressive and non-inflammatory architecture of the surrounding tissue [102,103]. The proposed framework targets this systemic barrier by seeking to ensure that tumor cell destruction takes place exclusively within an inflamed, cell-dense, and cytokine-rich environment.

Under this hypothesized model, the targeted destruction of resident metastatic lesions is mediated by the coordinated influx of activated host NK cells, M1-polarized macrophages, and allogeneic-driven cytotoxic effectors. Activated NK cells are conceptualized to infiltrate metastatic tumor lesions following each sequential intradermal/intravenous (ID/IV) administration cycle. Because many human solid tumors downregulate surface MHC class I expression to evade classic cytotoxic T-lymphocyte (CTL) recognition, engaging host NK cells during the early phases of the cascade provides a distinct mechanical pathway [51]. Unlike adaptive T cells, NK cells eliminate malignant targets without prior antigen presentation or MHC restriction. Thus, the early cytolytic function of infiltrating NK cells is hypothesized to initiate the ISV effect by mediating tumor membrane rupture via the directional exocytosis of lytic granules containing perforin and granzymes [104].

This cytolytic interaction is designed to trigger an ICD cascade that liberates intracellular tumor mutation-associated neoantigens (MANA) complexed with endogenous cellular chaperones—specifically heat shock proteins (HSPs) such as gp96, HSP70, and HSP90—alongside key damage-associated molecular patterns (DAMPs), including mitochondrial DNA, HMGB1 protein, extracellular ATP, and surface-exposed calreticulin [105]. Under these inflammatory ISV conditions, local host antigen-presenting cells (APCs) are recruited into the core of the damaged tumor lesions. Elevated IFN-γ signaling within the TME activates these infiltrating APCs, primarily DCs, which are essential for driving downstream adaptive anti-tumor immunity [106]. Notably, host DCs activated in the presence of concentrated, localized IFN-γ can also mediate direct tumoricidal ICD in a manner analogous to NK cells, providing an additional self-amplifying mechanism to support the localized in situ vaccine effect [107].

In this inflamed, IFN-γ-rich microenvironment, these mature host DCs internalize the freshly released HSP-MANA complexes via receptor-mediated endocytosis. Concurrently, the local DCs upregulate their expression of Interleukin-12 (IL-12), Interleukin-18 (IL-18), and critical B7-family co-stimulatory molecules (CD80/CD86), optimizing the physiological conditions for high-affinity tumor-specific priming [108]. This specialized pathway facilitates the efficient cross-presentation of the resident tumor epitopes onto host HLA class I and class II molecules. By presenting these previously hidden or tolerated neoantigens alongside strong allogeneic danger signals, the framework is conceptualized to override pre-existing peripheral tolerance. These licensed host DCs subsequently mature into definitive IL-12+ DC1 phenotypes and traffic to draining lymph nodes to prime a de novo, polyclonal adaptive immune response directed against the patient’s active metastatic variants [109], seeking to restore the durable immune-mediated tumor equilibrium lost during clinical progression.

Traditional cancer vaccines often fail to elicit a strong anti-tumor clinical response despite increasing tumor-specific T-cell titers, largely because resulting host lymphocytes cannot recognize tumor clones that downregulate MHC class I molecules [108]. The proposed model seeks to circumvent this escape mechanism through subsequent treatment cycles. The sustained presence of concentrated IFN-γ in the TME derived from activated allo-specific Th1 cells upregulates tumor MHC class I expression [110,111], rendering previously altered clones vulnerable to host T-cell recognition. Finally, the resulting tumor-specific CTLs in circulation can be non-specifically activated by the systemic wave of cytokines (IFN-γ, IL-12, and IL-18) released upon subsequent IV infusions via a bystander activation mechanism, accelerating their trafficking into the tumor lesions to execute tumor clearance [112].

### 2.5. Modulation of Tumor Immunoavoidance and Macroenvironmental Stabilization

In contrast to immune-enhancing protocols that can inadvertently amplify a compromised immune circuit, the proposed framework focuses on the deliberate immunomodulation of the systemic and local cellular balance between pro-tumor and anti-tumor populations [113], aiming to directly target tumor-induced immune escape mechanisms. Within this framework, the strategy of expanding the pool of circulating host memory Th1 cells, and subsequently driving their trafficking into metastatic sites, is conceptualized to amplify localized interferon-gamma (IFN-γ) concentrations within tumor lesions. Sustained IFN-γ signaling within the tumor microenvironment (TME) is hypothesized to facilitate tumor elimination by counter-regulating the functions of suppressive immune cell subsets, most notably regulatory CD4+ T cells (Tregs) [114,115], myeloid-derived suppressor cells (MDSCs) [116], and M2-polarized tumor-associated macrophages (TAMs) [117]. Furthermore, the concurrent upregulation of surface MHC class I expression on malignant cells—mediated by this localized IFN-γ influx—is hypothesized to counter tumor immunoavoidance, rendering malignant subclones vulnerable to cytotoxic T-lymphocyte (CTL) identification and subsequent cell-mediated lysis.

Finally, the framework incorporates a maintenance dosing protocol consisting of sequential monthly allogeneic Th1 cell administrations. This design feature is hypothesized to sustain the inflammatory state of the TME over an extended duration, providing continuous counter-regulation of adaptive tumor immunoavoidance and mitigating the re-establishment of immunosuppressive stasis. This sustained immunomodulatory window is conceptualized to support the extended survival signals and tracking metrics, such as Restricted Mean Survival Time (RMST), observed in emerging clinical evaluations. By continuously resetting the immunological macroenvironment away from a tolerogenic Th2 state and maintaining an inflamed, Type 1 stroma, this non-engineered approach aims to sustain active immunosurveillance against evolving variant clones, with the objective of translating a transient cytolytic interaction into a sustained clinical response.

### 2.6. The Adaptive Transition, Bystander Extravasation, and Polyclonal Memory Generation

The final stage of the proposed operational cascade involves a time-dependent transition from an initial, innate host NK cell-mediated tumor lysis to a sustained, tumor-specific adaptive CTL-mediated clearance. Under this theoretical model, the early waves of host NK cell infiltration and subsequent in situ vaccination are conceptualized to challenge peripheral tolerance mechanisms by releasing a comprehensive array of chaperoned tumor antigens directly into an inflamed, adjuvanted stroma. As host conventional DC1s cross-prime naïve T cells within the draining lymph nodes, a customized, polyclonal population of de novo tumor-specific memory CD8+ T cells is hypothesized to enter the peripheral circulation, joining the pre-established pool of circulating host allo-specific Th1 memory cells.

Concurrently, upon the administration of subsequent intravenous (IV) infusions of the living allogeneic cell formulation, the resulting systemic T-cell cytokine release is hypothesized to trigger a multi-wave vascular extravasation event. This systemic Type 1 cytokine surge is designed to drive non-specific bystander activation [112], upregulating LFA-1 and VLA-4 integrin complexes on both the host allo-specific Th1 memory cells and the newly generated, host tumor-specific memory CTL populations. Stimulated by the concurrent overexpression of endothelial cell-adhesion molecules (ICAM-1 and VCAM-1) [118], both effector cohorts are hypothesized to extravasate across the remodeled tumor vasculature, trafficking in tandem directly into the core of the metastatic solid lesions.

As the tumor microenvironment becomes progressively infiltrated by these cellular cohorts, the local concentration of Th1-derived and NK-derived interferon-gamma (IFN-γ) is hypothesized to reach a sustained signaling threshold. This sustained IFN-γ signaling alters the transcriptional profile of the solid tumor stroma and is hypothesized to stimulate the upregulation of surface MHC class I expression on previously altered, immunoedited cancer cells [108]. By restoring surface antigen presentation on variant clones that had historically escaped immunosurveillance, this persistent remodeling is conceptualized to provide the newly arrived, tracking tumor-specific CTLs with a localized effector mechanism [110]. By shifting the primary cytolytic burden away from non-specific innate lysis and into a high-affinity, MHC-restricted adaptive response, this non-engineered approach is hypothesized to mitigate tumor antigen escape, establishing a polyclonally diverse memory response capable of targeting metastatic disease and mitigating long-term recurrence [111,112].

## 3. Preclinical and Clinical Grounding of the Instructive Paradigm

### 3.1. Verification of Macroenvironmental Remodeling and Treg Subversion

The primary requirement of the proposed framework is the capacity of non-engineered allogeneic Th1 cells to reverse systemic, tumor-induced Type 2 (Th2) immune bias and break peripheral tolerance. In syngeneic murine models of established leukemia (BCL_1_) and melanoma (B16), weekly intradermal administrations of non-engineered allogeneic Th1 elements—acting as an active cellular adjuvant—successfully shifted host anti-tumor responses from a progressive Th2 baseline to a protective Type 1 (Th1) phenotype [86]. This immunomodulatory activity was strictly dependent on the pre-activated phenotype of the donor cells, as non-activated allogeneic lines failed to alter host polarization [88].

Mechanistic evaluations utilizing interferon-gamma receptor knockout (IFN-γR^−/−^) models demonstrated that this macroenvironmental shift is driven by physiological levels of donor-derived IFN-γ, which directly restricts the capacity of TGF-β-secreting tumors to convert naive host cells into immunosuppressive inducible regulatory T cells (iTregs) [115]. Beyond suppressing de novo Treg generation, exposure to this allogeneic Th1 cytokine cascade significantly reduced the suppressive capacity of pre-existing naturally occurring Tregs (nTregs) while rendering conventional host effector T cells non-responsive to Treg-mediated inhibition [115].

Furthermore, this Type 1 cytokine milieu rapidly licenses immature host dendritic cells into tumoricidal killer dendritic cells (KDCs) via the upregulation of surface CD40, CD86, and inducible nitric oxide synthase (iNOS) expression [107]. These licensed host KDCs execute direct tumor cell lysis via nitric oxide production, subsequently capturing liberated intracellular tumor mutations and migrating to regional lymph nodes to complete the operational loop of in situ vaccination [107] (see Table 2 for detailed mapping parameters).

### 3.2. Human Translation and the Survival-Radiological Progression Paradox

The human translational validity of this allogeneic instructive cascade has recently been evaluated in clinical trials involving both healthy immunosenescent cohorts and patients with advanced solid malignancies [3,20,119,120,121]. In a multi-center Phase 2B trial evaluating this dual-route allogeneic Th1 regimen in heavily pretreated, third-line microsatellite stable (MSS) metastatic colorectal cancer (mCRC)—a disease phenotype characteristically resistant to standard immune checkpoint blockades and with a median survival of 4–6 months—the therapy demonstrated an encouraging median overall survival (mOS) signal of 16.4 months [3].

This clinical signal correlated with a programmatic systemic shift, marked by the seroconversion of treated patients from baseline Interleukin-12 (IL-12) negativity to sustained IL-12 positivity alongside a concurrent rise in circulating soluble heat shock protein-70 (HSP-70) levels, which may reflect ongoing host-mediated tumor cell turnover in vivo. Because endogenous IL-12 is a classic, heterodimeric cytokine produced predominantly by innate myeloid subsets—specifically licensed dendritic cells and activated macrophages—it is typically undetectable in the peripheral circulation of late-stage metastatic patients due to profound tumor-induced immunosuppression and myeloid dysregulation. Consequently, this sustained systemic seroconversion suggests successful macroenvironmental immunomodulation, indicating that the circulating cytokine pool does not originate directly from the T-cell-based allogeneic therapeutic formulation itself, but rather may reflect a functional restoration of host cellular immunity driven by endogenous antigen-presenting cells. This distinct physiological separation supports the hypothesis that non-engineered donor cells operate primarily as an upstream instructive catalyst to engage host innate infrastructure, an observation consistent with the correlation between IL-12 positivity and extended survival reported in a prior Phase I/II clinical trial of 42 refractory metastatic solid tumor patients [122].

A defining feature of this stroma-remodeling cellular platform is a pronounced survival-radiological discordance in the Phase IIB clinical trial [3]. While the intent-to-treat population achieved an extended mOS of 16.4 months and a durable survival plateau confirmed by Restricted Mean Survival Time (RMST) analysis, 89.5% of radiologically evaluable patients met strict RECIST 1.1 criteria for progressive disease at Day 119 due to significant increases in target lesion diameters and the appearance of new radiographic nodules [3,119]. Crucially, this volumetric expansion occurred in the absence of clinical deterioration, with patients maintaining stable ECOG performance statuses. Because advanced MSS mCRC lesions are natively immunologically “cold” and non-inflamed, this survival-radiological paradox suggests that the volumetric increases captured on conventional imaging do not definitively represent therapeutic failure or true malignant proliferation. Instead, consistent with the hypothesized multi-phase model, this swelling may reflect a dense host leukocyte influx, localized edema, and distributed tumor necrosis triggered as the lesions actively transition from an uninflamed to an inflamed phenotype.

### 3.3. Synergy with Immune Checkpoint Inhibition and Reversal of Target Escape

This clinical hypothesis is further supported by observations where the administration of subsequent, short-course immune checkpoint inhibitors (ICIs) following allogeneic Th1 priming resulted in rapid, objective partial responses by RECIST 1.1 criteria, demonstrating substantial tumor debulking in an otherwise checkpoint-refractory malignancy [119]. This synergistic activity suggests that the non-engineered allogeneic instructive cascade alters the immunosuppressive stroma of human solid lesions in vivo, establishing a highly infiltrated microenvironment primed for therapeutic recognition.

Additionally, the sustained presence of concentrated IFN-γ within the tumor microenvironment upregulates surface MHC class I expression on previously altered, immunoedited cancer cells [110,111]. By restoring surface antigen presentation on variant clones that had historically escaped immunosurveillance, this persistent remodeling provides newly arrived, tumor-specific CTLs with a localized effector mechanism, aiming to override tumor antigen escape and imprint a polyclonally diverse host memory response capable of targeting metastatic disease.

Drawing definitive mechanistic conclusions regarding population-wide checkpoint responsiveness from a single-patient microsatellite stable metastatic colorectal cancer (MSS mCRC) clinical vignette carries inherent selection and statistical reporting biases. While the observed post-priming response following immune checkpoint inhibition (ICI) introduction provides critical insight into macroenvironmental modulation, this outcome must be interpreted strictly as a prospective, hypothesis-generating clinical signal. Determining whether this synergistic phenotype translates consistently across broader, heterogeneous patient populations remains an essential objective for future randomized clinical designs.

## 4. Mechanistic Limitations, Anatomical Barriers, and Biological Paradoxes

This proposed model must be explicitly balanced against historical failures observed in similar immunotherapeutic configurations. Specifically, clinical trials executing direct systemic administrations of recombinant IL-12 or IFN-γ historically encountered severe, occasionally fatal systemic toxicities or induced rapid, compensatory immune shutdown without therapeutic efficacy. Furthermore, early trials utilizing un-activated allogeneic leukocyte infusions frequently reported a complete lack of tumor regression due to the rapid, silent clearance of donor lines without the generation of an inflammatory cytokine surge. These contradictory findings highlight a critical biological limitation: if the allogeneic input lacks the necessary pre-activated phenotypic density, or if the host’s macroenvironment initiates rapid neutralizing clearance before bystander extravasation can materialize, the framework will reproduce the therapeutic failures of historical allogeneic designs. Furthermore, the model’s reliance on stable and sustained host Th1 polarization faces profound biological constraints within established solid tumor microenvironments. Chronic, long-term exposure to persistent somatic tumor antigens natively drives infiltrating host leukocytes toward advanced functional exhaustion, marked by the sustained upregulation of inhibitory surface receptors such as PD-1, TIM-3, and LAG-3. If the host macroenvironment collapses into an exhausted or mixed Th1/Th2 state under the pressure of continuous malignant exposure, the efficiency of the proposed instructive cascade will be severely constrained, stalling the crucial transition to high-affinity adaptive clearance and reinforcing the baseline therapeutic boundaries of the platform.

Consequently, a fundamental risk remains that the induced immune response may manifest merely as a non-specific, transient local tissue inflammation rather than generating a targeted, tumor-specific adaptive response. Because the initial phase of the cascade relies entirely on innate NK activation and non-specific bystander extravasation driven by an HvG mismatch reaction, generating sufficient antigen specificity against scarce, patient-specific somatic mutations remains a significant biological hurdle. If the host immune system treats the intervention purely as an isolated inflammatory tissue clearance event without initiating efficient, high-affinity DC cross-priming, the cascade will fail to imprint a durable adaptive memory repertoire, leaving the patient vulnerable to rapid disease recurrence once the transient cytokine wave subsides.

### 4.1. Fibrotic Desmoplasia and Localized Stromal Barriers

A primary structural limitation relates to the physical architecture of the tumor stroma, particularly in patients subjected to multiple prior lines of cytotoxic therapy. Advanced solid tumors frequently exhibit a dense, desmoplastic stroma characterized by an overproduction of extracellular matrix components and cross-linked collagen bundles [57]. This structural barrier can be compounded by prior localized ionizing radiation and multi-agent systemic chemotherapies, which induce chronic tissue stress, cellular senescence, and localized hypoxia, acting as drivers for progressive fibrosis.

Under the proposed model, systemic intravenous rechallenge relies on the upregulation of endothelial integrins to facilitate transendothelial migration [98]. However, even if circulating host allo-specific Th1 cells and natural killer (NK) cells execute endothelial adhesion, their migration into the core of metastatic lesions can be restricted by this fibrotic scar tissue. This desmoplastic encapsulation risks restricting the activated host infiltrate to the peritumoral periphery—a state of immunologic exclusion. This spatial separation may hinder physical contact with malignant cells, dampening the subsequent immunogenic cell death cascade required to facilitate in situ vaccination.

### 4.2. Anatomical Heterogeneity and Anisotropic Traffic Barriers

A second parameter restricting the universal application of this platform is the anatomical site of metastasis, with peritoneal disease, malignant ascites, and central nervous system (CNS) lesions representing distinct compartmental trafficking barriers. The proposed bystander extravasation mechanism relies on a functional, vascularized tumor-endothelial interface to direct activated memory cells from the systemic circulation into solid parenchymal tissue beds, such as the lungs, bone, or retroperitoneal lymph nodes [97]. Conversely, peritoneal metastases and floating malignant cell aggregates within ascitic fluid operate under altered fluid-dynamic and immunological conditions. In peritoneal carcinomatosis, tumors frequently seed along mesothelial surfaces that lack high-density organized microvasculature, or they survive as free-floating spheroids suspended within a protein-rich fluid macroenvironment. This fluid suspension creates an elevated interstitial fluid pressure (IFP) gradient that counteracts outward intravascular filtration. Consequently, activated host T cells and NK cells in the systemic circulation may fail to tether or exit the blood vessels into these fluid compartments due to a deficiency in localized endothelial cues. Furthermore, ascitic fluid can act as a physical sink, diluting donor-secreted and host-induced Type 1 cytokines, thereby potentially preventing the localized cytokine concentration threshold required to drive native NK-mediated membrane rupture or to induce killer dendritic cell differentiation in situ.

Parallel compartmental challenges emerge when considering intracranial metastases or primary CNS malignancies. While the intact blood–brain barrier (BBB) restricts the passage of passive macromolecules and non-activated resting leukocytes, activated effector immune cells possess the specialized adhesion molecules and chemokine receptors necessary to traffic across the blood-tumor barrier into the CNS parenchyma. However, this capacity for immune cell infiltration carries localized physiological risks. Depending on the volumetric size and baseline anatomical location of the intracranial tumor, a robust influx of activated host leukocytes—coupled with the multi-phase inflammatory cascade outlined in this model—may trigger localized, non-compliant vasogenic edema. Within the rigid constraints of the cranium, such inflammatory swelling can result in an adverse mass effect, potentially accelerating intracranial pressure, midline shift, or neurological deterioration. Consequently, while the platform’s mechanism remains theoretically capable of targeting CNS lesions via active effector trafficking, the clinical deployment of this cellular living adjuvant in patients with significant intracranial disease burdens may necessitate careful patient selection, proactive prophylactic management, or concurrent anti-inflammatory modulation to mitigate these localized volumetric risks.

### 4.3. The Interferon-Gamma Paradox: Checkpoint Activation vs. IL-12 Effector Override

The proposed model relies on sustaining elevated localized concentrations of Th1-derived and NK-derived IFN-γ within the tumor microenvironment to remodel the stroma, upregulate surface MHC class I expression, and drive the endogenous IL-12/DC1 axis. However, this hyper-inflammatory cytokine loop introduces a distinct biological paradox. Chronic exposure to physiological levels of IFN-γ serves as a primary homeostatic trigger for the transcription and surface expression of PD-L1 on malignant cells and Indoleamine 2,3-dioxygenase (IDO-1) within host myeloid cells [114]. This feedback loop represents an evolutionary self-defense mechanism designed to limit runaway tissue damage. In an oncological setting, this means that while the allogeneic flare facilitates host cellular infiltration, it simultaneously upregulates inhibitory checkpoints within the tumor stroma, presenting a potential mechanism for adaptive clonal compensation.

Crucially, however, emerging immunology literature demonstrates that this checkpoint activation does not uniformly result in functional T-cell paralysis if a highly coordinated, Type 1 cytokine macroenvironment is maintained. While acute IFN-γ drives the upregulation of surface PD-L1 molecules, the concurrent, sustained presence of high-density Interleukin-12 (IL-12)—synthesized and secreted by the newly licensed host DC1 or killer dendritic cell networks—exerts a dominant, counter-regulatory force. Mechanistically, IL-12 signaling through the IL-12Rβ1/β2 receptor complex on host lymphocytes acts to downregulate PD-1 receptor expression on infiltrating cytotoxic CD8+ T cells and natural killer (NK) cells. This persistent IL-12-driven stimulus preserves the transcriptional architecture of Type 1 effectors, maintaining robust STAT4 phosphorylation, optimizing intracellular granzyme B and perforin synthesis, and allowing cells to execute cytolytic tumor destruction despite the presence of surface-expressed PD-L1.

This molecular override mechanism provides a clear biological rationale for the distinct survival-radiological discordances documented across clinical configurations of this non-engineered allogeneic framework. In a multi-center Phase I/II clinical trial encompassing 42 heavily pretreated, refractory metastatic solid tumor patients with a variety of indications, the administration of intentionally mismatched, CD3/CD28-activated allogeneic memory CD4+ cells yielded a 0% conventional objective response rate yet demonstrated pronounced survival extension and immune-mediated tumor debulking without GvHD toxicity. Multi-parameter analyses identified serum IL-12 seroconversion as a definitive predictor of enhanced survival; 50% of the heterogeneous cohort transitioned to an active IL-12+ state, achieving a median overall survival of 211 days compared to 131 days for the IL-12- non-responder cohort (*p* < 0.009).

The reproducibility of this IL-12-mediated survival benefit is further highlighted by sub-cohort tracking within the same study. Among 16 refractory metastatic breast cancer (mBC) patients evaluated, 100% of the aggressive HER2+ subset (5 of 5) achieved robust IL-12+ seroconversion, generating a highly extended median overall survival of 416 days compared to only 134 days for the 11 HER2- patients (*p* < 0.05) [122].

Because Her2+ solid tumors are classically more aggressive and correlate with a poorer historical prognosis in late-stage salvage environments, this enhanced clinical response suggests that the allogeneic framework successfully harnesses host-donor incompatibility to drive a potent IL-12-driven hyper-inflammatory zone. This localized cytokine density permits newly cross-primed host effector repertoires and innate networks to override adaptive checkpoint barriers, driving a state of durable, immune-mediated tumor equilibrium rather than immediate macro-structural tumor regression. Ultimately, while this non-engineered allogeneic framework possesses the autonomous capability to maintain long-term cytolytic containment via this IL-12 override mechanism, the underlying checkpoint activation profile supports the rationale for a sequential combination approach utilizing secondary immune checkpoint inhibitors (ICIs) to fully maximize the therapeutic response.

### 4.4. Vascular Heterogeneity and Tissue-Specific Endothelial Exclusion

The practical execution of the bystander extravasation mechanism operates on the assumption of a uniform, vascularized tumor-endothelial interface across all structural metastatic niches. However, human metastatic malignancies display extensive physiological heterogeneity and tissue-specific microvascular phenotypes. In secondary sanctuary environments protected by restrictive physical boundaries—such as central nervous system metastases shielded by the blood–brain barrier or poorly vascularized osteolytic bone matrix beds—systemic endothelial activation loops may prove structurally insufficient to drive host effector leukocyte entry. Because the peripheral wave of circulating IFN-γ and TNF-α relies on a highly responsive, localized microvasculature to precipitate ICAM-1 and VCAM-1 overexpression, avascular or immunologically privileged niches can remain completely insulated from the systemic bystander influx. Consequently, while standard parenchymal visceral metastases undergo robust inflammatory infiltration, these specialized tissue sanctuary sites may structurally exclude the host cell infiltrate, creating local pockets of unchecked tumor antigen escape.

### 4.5. Immunological Crowding and Competitive Processing Kinetics

A notable biological friction point within the multi-phase cascade relates to the competitive processing mechanisms governing host antigen-presenting cells (APCs) during synchronized antigen presentation events. Throughout the localized dermal training and subsequent intra-tumoral lysis phases, host conventional type 1 dendritic cells (cDC1s) are heavily inundated with massive titers of foreign donor-derived cellular fragments following host-mediated allo-rejection. Concurrently, the cytolytic destruction of malignant targets liberates a significantly lower copy number of patient-specific somatic mutated neoantigens (MANA). Because receptor-mediated endocytosis and intracellular phagolysosomal processing machinery are saturable, this stark quantitative asymmetry introduces a latent liability of “immunological crowding”. If host cDC1s preferentially process and present the dominant pool of foreign mismatch HLA peptides over native, private tumor neoepitopes, the critical cross-priming threshold required to expand high-affinity anti-tumor CD8+ T cells could be structurally suppressed, requiring careful kinetic evaluation of host dendritic cell licensing parameters.

Furthermore, a major uncertainty remains regarding whether sufficient antigen specificity can be effectively generated during the early innate phase to ensure durable adaptive memory or whether the resulting immune response manifests broadly as localized tissue inflammation rather than a tumor-specific targeted response. However, it must be determined whether a stable and sustained Th1 polarization can be realistically maintained in vivo. In many established tumor microenvironments, chronic antigen exposure frequently leads to advanced host T-cell exhaustion or a mixed Th1/Th2 state, factors that could severely constrain the efficiency of the proposed cascade and stall transitions between intermediate phases.

A major mechanistic uncertainty within this framework relates to the exact temporal coordination governing the transition from acute innate rejection to adaptive host cross-priming. Current cross-species evidence remains inadequate to conclusively map the optimal kinetic window linking these distinct immunological phases. In real biological systems, if the host-mediated clearance of the donor cells proceeds too rapidly, the intravascular Type 1 cytokine wave may decay before sufficient host bystander cells can exit the vasculature and execute local tumor lysis. Conversely, if the temporal coordination between innate NK-mediated ICD and host DC migration into the regional lymph nodes is out of sync, the released somatic mutations will undergo rapid extracellular degradation before efficient cross-presentation can occur, breaking the necessary bridge to adaptive immunity.

### 4.6. The Iterative Priming Ceiling and Neutralizing Antibody Dynamics

While the therapeutic protocol functions by repeating iterative intradermal-intravenous (ID-IV) cell cycles to maximize circulating memory reservoirs, long-term repeated exposure is governed by strict homeostatic counter-regulation designed to prevent runaway systemic immunopathology. Chronic exposure to the identical foreign human leukocyte antigen haplotype may eventually precipitate advanced phenotypic exhaustion, anergy, or activation-induced cell death within the host’s anti-allo memory pool. Furthermore, continuous allogeneic rechallenge carries the risk of expanding host regulatory T-cell (Treg) loops or stimulating the systemic seroconversion of neutralizing anti-donor antibodies. If high-titer neutralizing host antibodies emerge, they can quickly bind, agglutinate, and eliminate the freshly infused intravenous donor formulation via complement-mediated or phagocytic pathways. This rapid intravascular neutralization prevents donor cells from executing structural cross-talk with circulating host memory elements, prematurely terminating the required systemic cytokine surge before bystander extravasation can materialize.

### 4.7. Translational, Operational, and Quality Control Challenges for Clinical Evaluation

Beyond the variables of desmoplasia, fluid compartments, and feedback loops, the clinical translation of this framework presents distinct biological, operational, and material challenges that define its current therapeutic boundaries.

#### 4.7.1. Radiological Dynamics and the Risk of Premature Treatment Discontinuation

In the supporting Phase 2B clinical evaluation, 31% of the intent-to-treat population discontinued the regimen prior to completing the third treatment cycle due to documented radiological progression [3]. While this phenomenon is hypothesized to reflect intense leukocyte infiltration and transient inflammatory swelling (‘pseudoprogression’), interpreting these volumetric adjustments requires caution. Crucially, because matching post-treatment longitudinal tissue biopsies and dynamic circulating tumor DNA (ctDNA) kinetic tracking were not systematically collected in early cohorts, it remains impossible to definitively differentiate benign inflammatory edema from true, unchecked malignant tumor proliferation or clonal antigen escape. If an expanding lesion represents true disease progression rather than immune infiltration, continuing therapy could inadvertently delay a patient’s access to alternative salvage modalities.

This operational hurdle underscores a major kinetic parameter of the platform: the temporal window required to successfully establish a high-titer host anti-allo Th1 memory bank via iterative intradermal injections can be radiologically obscured by early inflammatory pseudoprogression. Misinterpreting this initial stroma-remodeling phase as therapeutic failure poses a distinct operational risk, potentially depriving patients of long-term overall survival benefits. Consequently, this translational reality highlights the necessity of implementing next-generation iRECIST protocols and requiring the maintenance of stable ECOG performance status as the primary clinical determinant for treatment continuation in future randomized trials, preventing the premature exclusion of potential long-term responders.

#### 4.7.2. Potential Risks of Paradoxical Hyperprogression

Beyond the diagnostic ambiguity of imaging scans, forcing a potent HvG inflammatory flare introduces an active biological risk of driving paradoxical hyperprogressive disease (HPD). Certain advanced solid tumor clones harbor functional IFN-γ receptors wired directly to pro-tumorigenic JAK/STAT pathways, which can inadvertently stimulate cancer stemness, accelerate somatic proliferation, or drive tissue remodeling if the host’s downstream cytolytic execution networks are sub-optimally engaged. Furthermore, prolonged macroenvironmental inflammation can expand host myeloid regulatory networks or upregulate compensatory checkpoint axes like IDO-1 and PD-L1, potentially cementing a state of absolute therapy refractoriness. Proactively defining these hyperprogressive cohorts through upfront genomic screening represents a critical safety requirement for future trial designs.

#### 4.7.3. Donor Cellular Specifications, cGMP Manufacturing, and Product Quality Control

Because the proposed framework relies on the deliberate and rapid host-mediated clearance of allogeneic cells within a highly compressed physiological window, long-term cellular persistence or extended phenotypic stability within the suppressive host microenvironment is not required. Furthermore, because these therapeutic cells are derived directly from the peripheral blood of healthy donors rather than expanded from continuous master cell banks, risks associated with long-term clonal drift or continuous epigenetic instability are avoided. However, the model remains fundamentally dependent on the short-term quality, activation state, and phenotypic consistency of the freshly manufactured allogeneic Th1 formulation at the precise moment of administration. Healthy donor T cells exhibit inherent biological variability, necessitating strict manufacturing and regulatory quality controls to guarantee uniform immunogenic potency.

The specialized cellular input analyzed in this translational schema comprises living, pre-activated, human leukocyte antigen (HLA)-disparate Th1 cells that are completely free of genetic engineering (known under the designation AlloStim^®^; Immunovative Therapies, Ltd.). To satisfy rigorous safety paradigms, the initial biological material is harvested from healthy human volunteers who undergo sequential medical eligibility checks at an authorized center in compliance with federal guidelines for human tissue donations (21 CFR Part 1271). These diagnostic steps mandate exhaustive clinical evaluations, donor history surveys (AABB DHQ v.4), and validated viral panels tracking blood-borne entities, including human immunodeficiency viruses, viral hepatitides, cytomegalovirus, and spirochete pathogens. Purified CD4+ precursor fractions are isolated from healthy donor buffy coats and shifted towards a Type 1 inflammatory state using a cGMP-compliant manufacturing workflow (21 CFR Parts 210 and 211). Prior to batch qualification, the cells are chemically coupled with magnetic microparticles supporting anti-CD3 and anti-CD28 monoclonals at a synchronized 1:1 structural density to provide constitutive stimulatory signaling.

To confirm the precise instructive capability of each manufacturing lot, batch-release protocols implement an ex vivo functional validation assay rather than relying on structural morphology alone. Harvested cell-culture supernatants are isolated and co-incubated with human monocytic indicator lines (THP-1). Release authorization strictly requires these cell-free supernatants to autonomously drive a reproducible upregulation of surface co-stimulatory elements (CD80) and provoke a robust, quantified wave of endogenous Interleukin-12 (IL-12) secretion from the target monocytes, validating the batch’s dynamic capacity to license host myeloid structures in vivo. Qualified batches are preserved at a concentration of 1 × 10^7^ cells/mL inside an electrolyte preservation matrix (PlasmaLyte A) supplemented with 5% albumin and 2% cryoprotectant (DMSO), followed by deep freezing in liquid nitrogen shipper arrays. Thawing occurs rapidly at the bedside without washing, yielding a post-thaw recovery rate exceeding 80% with an immutable effector surface phenotype (IFN-γ+, CD4+, CD45RO+, CD62L^lo^, CD40L^hi^, CD25+). Because this approach avoids synthetic editing loops, viral integration vectors, or bespoke autologous cleanroom expansion, production costs are minimized by several orders of magnitude, providing a scalable, immediate therapeutic agent that enables flexible, multi-dose point-of-care clinical distribution.

#### 4.7.4. Mechanistic Divergence and Safety Profile of the Intravascular Rejection Flare

A critical question regarding the clinical translation of this framework is why the repeated intravenous (IV) infusions of living allogeneic Th1 cells into an already allo-primed host do not trigger the severe, life-threatening toxicities associated with Cytokine Release Syndrome (CRS). While both phenomena manifest as elevated systemic cytokine gradients, they are governed by completely separate cellular and molecular kinetics. True clinical CRS—classically observed following the administration of engineered cellular platforms or bi-specific T-cell engagers—is primarily a monokine-driven, positive feedback loop. In that setting, the continuous, autonomous in vivo expansion of the engineered lines hyper-activates host monocytes and macrophages, triggering an uncontrolled, self-amplifying cascade of pyrogenic monokines, most notably Interleukin-1 (IL-1) and Interleukin-6 (IL-6) [92,93].

In contrast, the intravascular rejection flare induced under this protocol represents a controlled, self-limiting, and strictly T-cell-driven immunological response. Because the recipient’s macroenvironment has been pre-conditioned via intradermal priming to harbor a high-titer bank of anti-allo memory elements, the subsequent intravenous rechallenge prompts immediate host-mediated recognition and tissue clearance. Rather than expanding autonomously, the non-engineered donor cells are cleared within a highly compressed physiological window. This rapid elimination halts the cellular cross-talk required to engage or hyper-activate host monocytes. Consequently, while the intravascular interaction successfully drives a transient, Type 1 T-cell cytokine surge (characterized by elevated IFN-γ and TNF-α levels) necessary to facilitate bystander cell extravasation, it lacks the sustained, monokine-mediated activation loop required to drive clinical CRS. Proactively defining this kinetic divergence explains the favorable safety profile observed across clinical cohorts, establishing a critical monitoring parameter for future randomized controlled trials.

Beyond the avoidance of acute CRS, a comprehensive safety analysis must consider broader risks associated with systemic inflammation, delayed autoimmunity, and off-target immune activation resulting from repeated allogeneic rechallenges. While the transient T-cell cytokine wave is structurally self-limiting, the prolonged systemic elevation of IFN-γ and TNF-α could theoretically trigger off-target immune activation against healthy tissues sharing structural homologies with the processed tumor antigens, presenting a latent risk of organ-specific auto-aggression. To mitigate these systemic inflammatory risks in clinical practice, future protocols must enforce mandatory pre-dose safety boundaries, including real-time monitoring of acute-phase reactants (e.g., C-reactive protein, ferritin) and the implementation of adaptive dosing algorithms or short-course corticosteroid mitigation protocols in the event of persistent, non-compliant systemic toxicities.

## 5. Conclusions and Future Outlook

The non-engineered, biology-driven cellular immunotherapy framework described in this article represents a conceptual shift in the approach to treating advanced, immunologically “cold” solid malignancies. Rather than continuing within an increasingly complex paradigm of multi-gene synthetic engineering developed to bypass or mask allogeneic cellular rejection, this model presents a mechanism whereby host-donor tissue incompatibility may be structurally leveraged as a high-potency, off-the-shelf immunomodulatory stimulus. By separating the clinical intervention into distinct anatomical stages—moving systematically from localized dermal priming to systemic vascular rechallenge—the framework utilizes endogenous host immune pathways evolved to clear foreign cells, correct systemic Type 2 helper T-cell biases, modulate local tumor-induced immunosuppression, and establish the inflammatory conditions required to facilitate an in situ vaccination effect.

Looking forward, advancing this macro-level immunological strategy from an empirical concept toward a highly predictable, precision-driven clinical development paradigm will be heavily propelled by the integration of advanced artificial intelligence and structural machine learning frameworks. While the proposed platform relies on the host’s native antigen-presenting cells to execute empirical in situ vaccination, emerging deep learning architectures—most notably the AlphaFold AI model and its latest molecular iterations [123]—offer a powerful structural foundation to decode these hidden cellular interactions.

By leveraging structural deep learning, investigators can accurately predict the three-dimensional conformations of newly primed host T-cell receptors (TCRs) interacting with their cognate patient-specific tumor neoantigens complexed with Human Leukocyte Antigen (peptide-MHC) molecules. These AI-empowered structural tools enable systematic evaluation of the immunogenicity of native tumor neoantigens ex ante, mapping the spatial architecture of immune recognition events and tracking the expansion of the host’s polyclonal anti-tumor memory repertoire over time. Integrating this atomic-resolution structural data with longitudinal clinical response registries provides a valuable, structure-guided roadmap for future research, allowing investigators to rationally select optimal donor tissue haptotypes, predict bystander activation efficacy, and engineer complementary therapeutic molecules designed to fully maximize precision-driven solid tumor clearance.

Empirical preclinical models and clinical observations across separate geriatric and oncology clinical trial populations provide an encouraging translational proof-of-concept for this non-engineered cellular alternative [20,86]. Longitudinal monitoring suggests that this iterative protocol can potentially modulate systemic immune seroconversion and stimulate tumor cell death directly at metastatic sites. This process is conceptualized to alter the suppressive stroma of non-responsive microsatellite stable tumors and prime a functional, polyclonal, and tumor-specific host adaptive T-cell repertoire. This phenomenon is hypothesized to contribute to reversing checkpoint refractoriness, potentially facilitating objective tumor responses following sequential immune checkpoint inhibition [119].

However, because full characterization of these complex downstream cellular interactions remains ongoing, establishing their broader clinical and statistical validity requires expanding beyond exploratory case reports and non-randomized single-arm designs. Consequently, there is an evident utility in initiating rigorous, large-scale randomized controlled trials (RCTs). Future randomized clinical evaluations should be designed to compare this iterative allogeneic approach against standard-of-care options in treatment-refractory settings, evaluating population-wide median overall survival benefits and Restricted Mean Survival Time (RMST) parameters while accounting for non-proportional hazard variations and transient pseudoprogression events inherent to inflammation-inducing cellular therapeutics.

The successful execution of future randomized trials depends on the simultaneous integration of a comprehensive, multi-layer biomarker tracking matrix capable of mapping the kinetic milestones of the allogeneic inflammatory cascade. Relying exclusively on conventional RECIST 1.1 criteria may be insufficient, as volumetric imaging methods are often structurally limited in assessing stroma-remodeling platforms due to the temporary confounding effects of benign host immune cell infiltration.

Future protocols should incorporate adapted immune-related response criteria, such as iRECIST, alongside detailed monitoring of downstream serum monokine and T-cell cytokine fluxes (including tracking of IL-12, IL-18, and IFN-γ). These measures should be paired with tissue-level biopsies to assess shifts in the local intratumoral ratio of cytotoxic effectors to regulatory populations. Furthermore, tracking systemic indicators of tumor membrane fragmentation—such as circulating cell-free DNA variants and longitudinal soluble heat shock protein dynamics—remains highly relevant to evaluate in vivo immunogenic lysis. By combining rigorous randomized trial architecture with objective biochemical and molecular biomarker arrays, clinical investigators can reliably evaluate whether leveraging allo-incompatibility for in situ immunization can provide a scalable solution for patients with advanced solid malignancies.

## Figures and Tables

**Table 2:** Cross-Species Proof-of-Concept Target Parameter Mapping.

Operative Mechanism Phase	Murine Proof-of-Concept (Syngeneic Models)	Human Translational Grounding (Published Datasets)	Diagnostic/Monitoring Parameter
Phase 1: Macroenvironmental Deviation	Shifting anti-tumor responses from a progressive Th2 baseline to a protective Type 1 phenotype in the BCL_1_ leukemia model [86,88].	Systemic immune rejuvenation and expansion of circulating IFN-γ+ Th1 elements in elderly cohorts [20].	Serum IL-12 seroconversion from negative baseline to sustained positivity.
Phase 2: Remodeling Tumor Defense Shield	Direct down-regulation of inducible Treg conversion metrics via Th1-derived IFN-γ in aggressive 12B1 leukemia models [115].	Elimination of checkpoint refractoriness in late-stage mCRC cohorts, yielding rapid partial responses upon sequential ICI re-challenge [119].	Sustained life expectancy plateaus tracked via non-parametric RMST metrics.
Phase 3: Bystander Effector Recruitment	Localized iNOS upregulation and dendritic cell licensing into tumoricidal killer dendritic cell (KDC) phenotypes [107].	Volumetric lesion enlargement and tissue density swelling captured via RECIST 1.1 dimensions in advanced solid tumors [119].	Pseudoprogression tracking utilizing updated iRECIST protocols in clinical cohorts.
Phase 4: In Situ Vaccination Loop	Complete regression and long-term protective immunity against lethal active tumor rechallenges [86].	Extended overall survival (16.4 months mOS) in heavily pretreated, third-line salvage MSS mCRC populations [3,119].	Longitudinal tracking of peripheral soluble HSP-70 concentrations.

## Data Availability

No new data were created or analyzed in this study. Data sharing is not applicable to this article.

## Abbreviations

AABB	American Association of Blood Banks
APC	Antigen-Presenting Cell
ATP	Adenosine Triphosphate
BBB	Blood–Brain Barrier
CAR	Chimeric Antigen Receptor
CCR5	C-C Chemokine Receptor Type 5
CD	Cluster of Differentiation
cDC1/DC1	Conventional Type 1 Dendritic Cell
CFR	Code of Federal Regulations
cGMP	Current Good Manufacturing Practices
CLIA	Clinical Laboratory Improvement Amendments
CMV	Cytomegalovirus
CNS	Central Nervous System
CRISPR	Clustered Regularly Interspaced Short Palindromic Repeats
CRS	Cytokine Release Syndrome
CTL	Cytotoxic T Lymphocyte
CTLA-4	Cytotoxic T-Lymphocyte-Associated Protein 4
CXCL	C-X-C Motif Chemokine Ligand
CXCR3	C-X-C Motif Chemokine Receptor 3
DAMP	Damage-Associated Molecular Pattern
DC	Dendritic Cell
DHQ	Donor History Questionnaire
DMSO	Dimethyl Sulfoxide
DNA	Deoxyribonucleic Acid
EBV	Epstein–Barr Virus
ECOG	Eastern Cooperative Oncology Group
FDA	Food and Drug Administration
GvHD	Graft-versus-Host Disease
GM-CSF	Granulocyte-Macrophage Colony-Stimulating Factor
HCT/Ps	Human Cells, Tissues, and Cellular and Tissue-Based Products
HBV/HCV	Hepatitis B Virus/Hepatitis C Virus
HER2	Human Epidermal Growth Factor Receptor 2
HIV	Human Immunodeficiency Virus
HLA	Human Leukocyte Antigen
HMGB1	High-Mobility Group Box 1
HPD	Hyperprogressive Disease
HSA	Human Serum Albumin
HSP	Heat Shock Protein
HTLV	Human T-Cell Lymphotropic Virus
HvG	Host-versus-Graft
ICAM-1	Intercellular Adhesion Molecule-1
ICANS	Immune Effector Cell-Associated Neurotoxicity Syndrome
ICD	Immunogenic Cell Death
ICI	Immune Checkpoint Inhibitor
IDO-1	Indoleamine 2,3-Dioxygenase 1
IFN-γ	Interferon-Gamma
IFP	Interstitial Fluid Pressure
Ig	Immunoglobulin
IL	Interleukin
iNOS	Inducible Nitric Oxide Synthase
iNKT	Invariant Natural Killer T
IPP	Isopentenyl Pyrophosphate
IRB	Institutional Review Board
ISV	In Situ Vaccine/In Situ Vaccination
IV	Intravenous
IVIG	Intravenous Immunoglobulin
JAK	Janus Kinase
KDC	Killer Dendritic Cell
LC	Langerhans Cell
LFA-1	Lymphocyte Function-Associated Antigen-1
LN_2_	Liquid Nitrogen
LNP	Lipid Nanoparticle
MAIT	Mucosal-Associated Invariant T
MANA	Mutation-Associated Neoantigen
mBC	Metastatic Breast Cancer
mCRC	Metastatic Colorectal Cancer
MDSC	Myeloid-Derived Suppressor Cell
MHC	Major Histocompatibility Complex
mOS	Median Overall Survival
MR1	MHC Class I-Related Protein 1
mRNA	Messenger Ribonucleic Acid
MSS	Microsatellite Stable
NGS	Next-Generation Sequencing
NK	Natural Killer
PD-1	Programmed Cell Death Protein 1
PD-L	Programmed Death-Ligand 1
pMMR	Mismatch Repair Proficient
PRR	Pattern Recognition Receptor
RCT	Randomized Controlled Trial
RECIST	Response Evaluation Criteria in Solid Tumors
RMST	Restricted Mean Survival Time
RNA	Ribonucleic Acid
scFv	Single-Chain Variable Fragment
STAT	Signal Transducer and Activator of Transcription
STING	Stimulator of Interferon Genes
TAA	Tumor-Associated Antigen
TAM	Tumor-Associated Macrophage
TCR	T-Cell Receptor
TGF-β	Transforming Growth Factor-Beta
Th1/Th2	Type 1 Helper T-Cell/Type 2 Helper T-Cell
TIL	Tumor-Infiltrating Lymphocyte
TIM-3	T-Cell Immunoglobulin and Mucin-Domain Containing-3
TLR	Toll-Like Receptor
TME	Tumor Microenvironment
TNF-α	Tumor Necrosis Factor-Alpha
Treg	Regulatory T-Cell
TSA	Tumor-Specific Antigen
USP	United States Pharmacopeia
VCAM-1	Vascular Cell Adhesion Molecule-1
VST	Virus-Specific T Cell
WNV	West Nile Virus
